# The dark side of technological advances in analysis of microbial ecosystems

**DOI:** 10.1186/s40104-019-0357-2

**Published:** 2019-06-17

**Authors:** Mick Bailey, Amy Thomas, Ore Francis, Christopher Stokes, Hauke Smidt

**Affiliations:** 1 Bristol Veterinary School, University of Bristol, Langford House, Langford, Bristol, BS40 5DU UK; 2 Laboratory of Microbiology, Wageningen University & Research, NL-6708 Wageningen, WE Netherlands

**Keywords:** Biobanking, Experimental design, Horizon-scanning, Microbiome, Replacement, Reduction, Refinement, Technological advances

## Abstract

Recent technological advances mean that samples from animal experiments may be analysed more cheaply, more easily and with a much greater return of data than previously. Research groups are frequently faced with a choice of continuing to use established technology in which they may have made a significant investment of time and resources, and have significant amounts of reference data, or switching to new technology where reference data may be limited. Apart from cost, the choice needs to be based on a comparison between the increase in data available from future experiments by switching and the value of comparison with reference data from historical experiments analysed with earlier technology. One approach to this problem is to ensure that sufficient quantity and variety of samples are taken from each experiment and appropriately stored to allow re-establishment of a sufficiently large reference set and to avoid the need to repeat animal experiments. The establishment of ‘biobanks’ of experimental material will require funding for infrastructure, consistent storage of metadata and, importantly, horizon-scanning to ensure that samples are taken appropriately for techniques which will become accessible in future. Such biobanks are a recognised resource in human medicine, where the value of samples increases as more analysis is carried out and added to the metadata.

## The pace of technological change

There has been considerable interest in the idea that the recent exponential growth in scientific publications and journals can be interpreted as a consequence of the drive for individual scientists to publish more, with the implication that there has been a decrease in methodological and analytical robustness of the research described [1, 2]. However, there are also, clearly, many other drivers for the increase, and it seems likely that the exponential rate of technological development is also a major contributor [3]. The rate of transfer of these technologies into routine research means that previously unanswerable scientific questions are likely to become increasingly accessible to interrogation. While providing enormous opportunities, this technological development also raises challenges. Two specific issues are the question of when to switch from an established methodology, where serial data sets from experiments are at least comparable, to the next generation techniques where reference values are absent; and, secondly, that it becomes likely that animal experiments may need to be repeated every few years in order to analyse the same outcomes but using the new technologies, with implications for the drive for replacement, refinement and reduction of the animals use in experiments (the 3Rs) [4]. Here, we will examine the impact of technological advances on, specifically, research on animal health and disease, and the implications for the way in which we should be developing our hypotheses, experimental designs, sample collection and analysis. While the considerations are likely to be broadly applicable, we will focus on the recent interest in establishing links between host-microbial ecosystems (the microbiome) and immune and metabolic systems.

For the purposes of this discussion, technology will be defined as the processes separating the development of a hypothesis from the acceptance, rejection or modification of that hypothesis. That is, technology will be taken to include the process of designing the experiment, carrying it out, collecting samples, extracting data from the samples and carrying out appropriate manipulation of the data to test the hypothesis or to develop predictive algorithms. Technological advances may be separated into two types: those which represent incremental advances in single steps within an overall technique (for example, the move from mercury to digital thermometers, or the use of bead-beaters in cell lysis for DNA isolation) [5]; and those which create a step-change in the process (the move from thermometers to remote sensing such as infra-red thermography or surface/internal thermistors, or from microarray to 16S ribosomal RNA (rRNA) gene sequencing for characterising the microbiome, or from Edman degradation to mass spectrometry for peptide sequencing) [6–8].

### What determines the move to new technology?

Laboratories and institutes frequently invest considerable effort in staff time or capital costs in implementing and validating particular technologies, with the result that there is usually significant resistance to switching to a new technology. Many factors will affect the decision to switch, but the main drivers are probably costs and the ability to extract novel or greater amounts of information.

#### Costs of new technologies

In many cases cost is one of the major determining factors. This can affect decisions in different ways. Initially, cost is usually a negative driver, where early adoption of technology is usually associated with extremely high costs of new equipment and often low reliability and expected rapid obsolescence. Later in the cycle of the technology, the cost of the new equipment often drops below that of the previous generation, affecting decisions to switch positively. In the main, the current structure of research funding requires either that institutes commit capital funding for the purchase; or that PIs obtain external capital funding for new equipment; or that funding for consumables covers the costs of subcontracting the processing of samples to a service. In many cases, funding streams are not adequate to cover the costs of the necessary equipment within research institutes and strategic decisions are made to subcontract. However, service subcontractors will, of course, include contributions towards necessary future equipment upgrades within their costs.

The choice of strategy (capital purchase of new equipment or reliance on service providers) depends very much on the costs of the equipment, and increasing uptake by research communities inevitably results in a decrease in price, such that strategic decisions may need to be re-considered with time. One of the first considerations, therefore, is the position in the development cycle: is the technology cutting edge and expensive or routine and cheap?

**Fig. 1:**
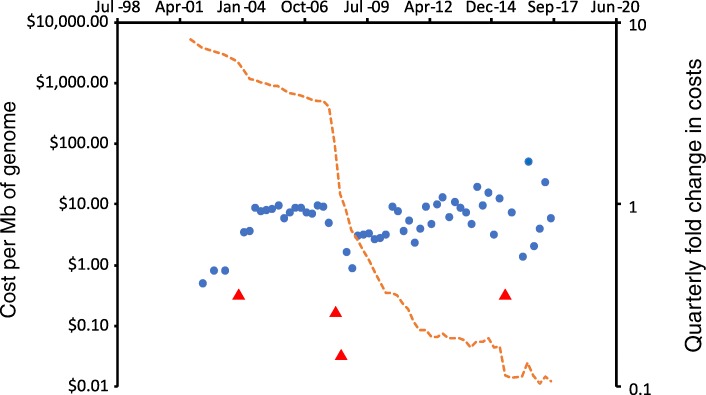
Costs of DNA sequencing over time. Orange line, costs of DNA sequencing, redrawn from data obtained from Wetterstrand [9]. Circles, quarterly fold change in costs (mean 0.85, SD 0.25): red circles indicate values greater than 2 standard deviations from the mean (log data)

Historical data on costs of processing samples are not readily available for most technologies. However, data on costs of sequencing have been maintained by the National Institutes for Health (NIH) for several years now [9] and show consistent, dramatic decreases, due in part to incremental improvements and economies of scale as more users adopt sequencing technology, and also as a consequence of step changes in sequencing technology (Fig. 1). The data on absolute and relative cost suggest such step changes in 2003, 2008 and 2015, although it should be noted that these are likely to be a consequence of a combination of economic and technical factors. Notably, costs have not consistently decreased over the last few years, and it will be interesting to see whether the trend does continue at the same pace, or whether costs of sequencing are approaching an asymptote.

#### Increasing delivery of data from experiments

The primary driver of the uptake of new technologies should be the ability of the new technique to deliver either a greater volume of data or more precise or reliable data. While extracting the maximum amount of data from an experiment should always be desirable, it carries several drawbacks. Firstly, the computing power necessary to analyse the increased volume of data will carry its own financial costs. Simple algorithms (the so-called Moore’s Law) suggest that computer power has doubled approximately every 2 years, but estimates are that this may slow down in the near future unless step-change technologies like quantum computing become widely available [10, 11]. Together with the phenomenon of software ‘bloat’, where an increasing amount of the available computer power is used in translating between layers of software or hardware compatibility and is unavailable to the user, this may even result in decreasing returns in new hypotheses or ideas from increasing amounts of data.

Secondly, there are issues over the availability of suitably trained staff to deal with the increased volume of data. As in the 1990s when trained molecular biologists were difficult to find, there are very few scientists now with appropriate experience in data analytics as well as sufficient background in agricultural science. As with molecular biologists, it is likely to be a decade or more before either such trained individuals become available or the interfaces to data analytics software become accessible to existing scientists.

## The problems of technological change for animal experiments

### The need to repeat experiments as technologies for sample analysis change

**Fig. 2:**
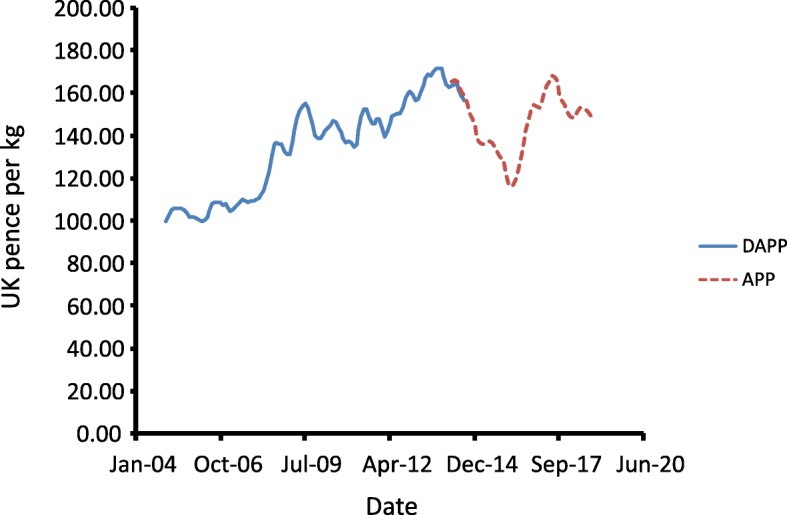
Costs of pig production in the UK (UK pence per kg). DAPP, deadweight average price; APP, average pig price. Source: MLC/AHDB pork (https://pork.ahdb.org.uk/prices-stats/prices/pig-prices-eu-spec/)

For many reasons, uptake of new technologies creates a number of obvious problems for animal experiments, particularly those involving large livestock species. Essentially, once an experiment is finished, there is no way to go back and re-take samples. If a new technology requires samples to be taken differently such as, for example, intestinal luminal samples into broth for culture-based techniques for analysis of microbiomes or snap frozen for DNA-based techniques, previous experimental designs may need to be repeated. Where experiments involved the use of animals, this is likely to carry a significant financial and ethical cost. The costs associated with production of large agricultural species suitable for animal experiments, and of carrying out experiments involving manipulation of groups of those animals, is extremely unlikely to decrease. While financial and political instability do contribute to livestock prices, the current trend in those countries where agricultural research is well-funded is, quite rightly, towards increasingly welfare-friendly production with associated increases in costs. This, plus the increasing costs of buildings and labour mean that prices for pigs, for example, have increased overall over the last 15 years (Fig. 2). With increasing pressure on the growth of agriculture also arising from concerns over impacts on climate [12], this trend is likely to continue.

### Decreasing backward compatibility of data from analysis of experimental samples

**Fig. 3:**
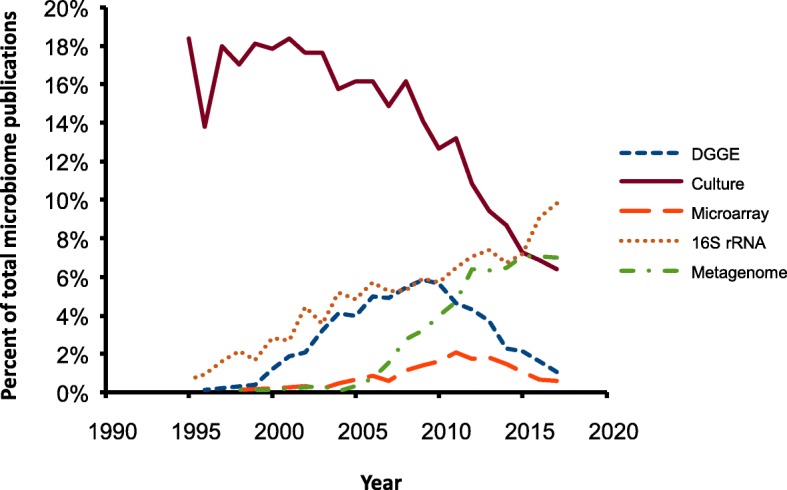
Publications on microbiome or microflora mentioning culture, DGGE, microarray, 16S rRNA sequencing or metagenomics. Source: Web of Knowledge (Clarivate Analytics), December 2018

It is also increasingly apparent that step changes in methodologies are associated with at least some level of obsolescence of data derived from previous experiments. A striking example of this is the change from assessing intestinal microbiomes using culture-based approaches during the 1990s to DNA-based approaches in the 2000s. Figure 3 shows the results of searches for microbiome-related publications which specifically mention either culture, denaturing gradient electrophoresis (DGGE), microarray, 16S rRNA sequencing or metagenomics between 1995 and 2017. Interestingly, the use of 16S rRNA sequencing was being reported before 2000 and its uptake has continued to rise consistently since then. Amplification of 16S rRNA genes was the basis for the widely used technique of DGGE, which began to be reported between 2000 and 2010 but has been in decline since then. In contrast, microarray-based approaches to microbiome analysis began to be reported in significant numbers from about 2005: while these approaches could also be based on 16S rRNA sequences, there is no absolute requirement for this and more recent arrays use operational taxonomic unit (OTU) specific sequences from whatever part of the genome provides the greatest specificity under the working conditions of the array. Despite this, and despite the higher dynamic range, reported usage of microarrays also seems to be declining. Finally, metagenomics-based publications have also increased consistently from 2005. Given that both 16S rRNA and metagenomics-based approaches seem to be consistently increasing and to be included in similar proportions of microbiome papers, it will be interesting to see whether one or other becomes dominant over the next 5–10 years. While it might seem that the obvious progression would be for metagenomics to supersede 16S sequencing, this may not necessarily be the case immediately, since the increasing availability of microbial whole genome sequences provides the opportunity for inferring metagenomes from 16S rRNA sequences, using tools such as PiCrust [13, 14]. However, in the longer term, as sequencing power and the ability to resolve closely related whole genomes increases, it may well ultimately become easier to infer full metagenomes to much greater resolution from partial metagenomes than from 16S rRNA sequences.

**Fig. 4:**
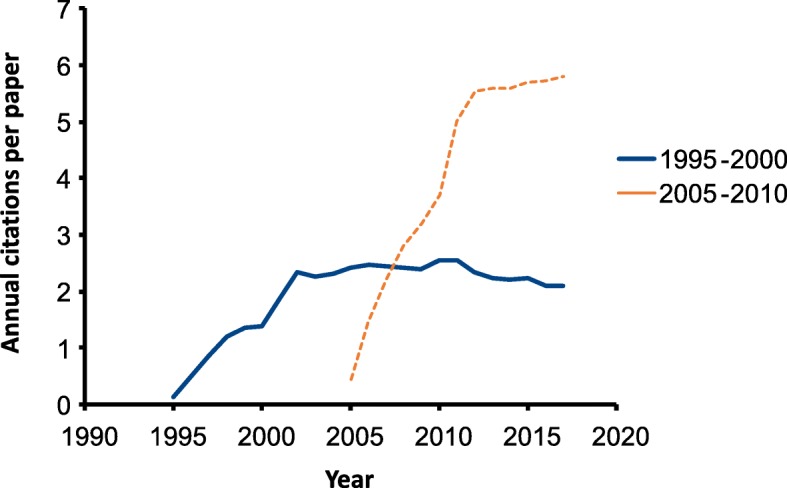
Annual citation rates for papers on microbiomes published between 1995 and 2000 (blue line), or between 2005 and 2010 (orange line). Source: Web of Knowledge (Clarivate Analytics), December 2018

The succession of techniques (culture to 16S-based to metagenome) raises questions as to the extent to which results obtained from experiments 5, 10 or 20 years ago may be interpreted against current experiments: should we reject data based on techniques which have now been superseded? While it may be argued that there is no a priori reason to reject conclusions based on culturing known groups of organisms from intestinal or faecal samples from experimental animals, we are now aware that the variation observed in these earlier experiments represents only the tip of the iceberg, and that significant differences between experimental groups or animals could have been present in the absence of culture differences [15]. For these reasons, results from microbiome experiments carried out prior to 2000 are not easily comparable to those after 2005. Interestingly, despite this, continuing citation rates for papers prior to 2000 are still not declining markedly and are comparable to those between 2005 and 2010 (Fig. 4), indicating that the scientific community still values the conclusions reached.

The succession of techniques described has created problems for groups engaged in microbiome research. Many laboratories have invested resources in establishing laboratory and bioinformatics pipelines which have rapidly become superseded by new developments, and need to consider the issue of whether to change. However, a further key consideration is that the accumulation over time of a large archive of samples analysed in exactly the same way provides an invaluable reference against which new samples or new experiments can be compared. Under these circumstances, even changing a very small component of the pipeline such as the DNA isolation kit, may make subsequent data difficult to compare with existing reference data [16, 17]. Adopting a completely new generation of technology mostly means that newly analysed samples must be assessed with minimal reference to previous results.

**Fig. 5:**
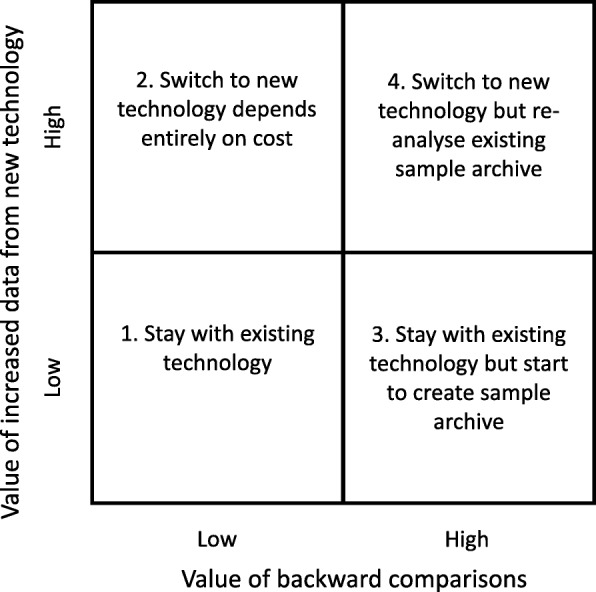
Decision square showing possible routes when considering changing to new techniques. The *Y*-axis represents the value to the understanding of experimental effects or to the ability to generate or test hypotheses. The *X*-axis represents the value of prior investment in existing technology, in particular the investment in samples from previous experiments

Under these circumstances, there may be considerable value in persisting in the use of a well-established pipeline rather than switching to new technology. Ultimately, the decision to make the switch depends on the amount of data or inference which can be derived from individual samples. We could consider the value of the sample to derive from three components: the data obtained by processing the single sample (which increases from DGGE through microarray and 16S rRNA to metagenomics); the inferences which can be made by comparing internally within a single, controlled experiment (e.g. the effect of a single probiotic under a defined set of circumstances); and the inferences which can be made by comparing an experiment with a pre-existing set of other experiments analysed in the same way (e.g. the robustness of ‘enterotypes’ in pigs across a large set of samples collected over time) [13]. The decision to stay with existing or switch to new technologies depends in part on the relative value of these three components. For simplicity, the problem may be considered as a simple decision square, where the value of the increased data from a new technology may be high or low, and the value of backward comparisons may also be high or low (Fig. 5). Where the value of both is low (that is, where the new technique currently offers very little increased data return, but there is relatively little investment in the results of previous techniques (Fig. 5 box 1), the decision should be based on horizon-scanning as to the future developments in both technologies. Once the new technology provides significantly greater data return, the decision is a matter of cost (Fig. 5 box 2). On the other hand, where investment in previous technology has been high, the initial response (Fig. 5 box 3) might be to begin archiving sample material for re-analysis such that, when data return from the new technology increases, it will be possible to re-analyse archived samples for backward comparisons (Fig. 5 box 4). A critical conclusion, then is that horizon-scanning and sample archives or biobanks are important for maintaining forward and backward compatibility, and these will be considered later.

### Hypothesis-driven and bias-free experimental design

In the majority of cases, experiments are designed to answer specific hypotheses or questions, even when the outcomes measured are highly dimensional, as in the case of intestinal microbiomes or host transcriptomes. Appropriate experimental design requires the necessary controls (positive, negative or both) and numbers of experimental units (cells, animals or groups) to allow the results to have a high probability of demonstrating that the hypothesis is false. Agricultural science has a long history of rigorous experimental design, power calculation and statistical evaluation [18]. However, the development of the new omics technologies is making statisticians increasingly aware of the potential for both measured and unmeasured variables to confound our results and to create unexpected differences between replicates.

Essentially, the unwritten assumption involved in testing hypotheses with specific experimental designs is that an analytical plan defining the statistical approach to be used should also be identified prior to data gathering. In many cases, the experimental design defines an appropriate statistical analysis. If statistical approaches are not pre-defined, then it becomes tempting to employ progressively complex data-transformation, data subsetting or analytical approaches, in the hope that one or more will identify an effect. This may consist of conducting either multiple comparisons using the same statistical test or employing a multitude of different statistical tests. In the first instance, it is likely that multiple comparisons will predispose to Type I errors, unless compensated for [19]. If experiments are then repeated, results which were apparently significant in the first replicate may not be observed in the second or subsequent replicates, leading to the apparent replicate effects seen in many agricultural experiments and to conclusions of poor reproducibility. In the case of multiple analytical approaches, it is entirely possible to apply different transformations or tests in a logical, “step-wise” manner for robust inference. However, if multiple different statistical approaches are used which actually address the same question, then conclusions may be reinforced by apparent concordance created because variables are not independent.

Both of these approaches – multiple comparisons and multiple analytical approaches - are entirely legitimate for data exploration and for generating hypotheses for further testing, but they cannot safely be regarded as tests of a pre-existing hypothesis. For this reason, much of the kind of exploratory data analytics being developed eschews the probability values which animal scientists are familiar with [20–22]. One solution to this is to design experiments and register not just the experimental design but also the analytical approach before the experiment is carried out [23]. Approaches of this sort, pioneered by the Alltrials initiative (http://www.alltrials.net/) and in development for veterinary clinical trials (https://vetalltrials.org/) help to clearly separate the analysis used to test the original hypothesis from that used to explore for possible further hypotheses. A second solution is to design experiments with sufficient redundancy in statistical power that the results can be separated into a training set, on which a range of analytical approaches can be tried and hypotheses developed, and a testing set on which the final options can be assessed. This latter approach has been most used in oncology studies where the results of many assays are used to detect tumours or predict outcomes [24]. Ideally, both of these require similar independent repositories: the first for pre-registering the experimental design and analytical approach; the second, for securing the testing dataset completely isolated from the statistician working with the training dataset. However, the latter approach will need experiments to be designed with sufficient numbers of replicates to allow redundancy in statistical power, increasing both the financial cost and the ethical considerations.

## Biobanking experimental samples for analysis of host-microbiome interactions

As the rate of development of technology accelerates, it will be important that the introduction of new methodologies does not mean that experiments need to be repeated unless absolutely necessary. Where experiments are designed to test a single hypothesis, it may be possible to take a single sample, carry out a single laboratory test and analyse the outcomes. Simplicity, staff availability and cost of the sample storage and analysis may make this option preferable. However, if this design is extended such that multiple laboratory analyses, or a single omics approach such as metabolomics, are carried out on the sample, then the data analysis is also likely to extend into the data exploration approaches outlined above, such that the experiment actually identifies a series of further hypotheses which then need to be tested. Under these circumstances, it begins to be important to take more samples from the initial experiment than had been originally envisaged. For example, the changes observed in a colonic microbiome after nutritional intervention might indicate effects upstream of the large intestine, in which case samples from the ileum or jejunum might be needed.

This last point is likely to become increasingly important and is something that could, potentially, be addressed now. As new technologies are developed and the costs of processing samples through existing technologies decreases, the costs of animal experiments will continue to rise. Under these circumstances, it would seem that taking many more samples than are immediately necessary provides a way of future-proofing our experiments, providing these samples are taken appropriately and stored with appropriate and adequate metadata. Essentially, this is an argument for routine biobanking of large numbers of samples and associated metadata from animal experiments, in such a way as to provide easy access to subsets of samples appropriate for testing specific, novel hypotheses. Such novel hypotheses could derive from prior analyses of subsets of the sample biobank or from the availability of new technologies. Thus, for example, where laboratories delayed the adoption of 16S rRNA gene sequencing for microbiome analysis in favour of microarray because a significant part of the value of each sample or experiment derived from comparison with a pre-existing dataset, the availability of biobanked faecal or intestinal samples would enable the re-creation of the pre-existing database when the cost of the new technology decreases to the point where re-analysing the samples becomes feasible.

Biobanks of appropriately taken, appropriately stored samples plus appropriate metadata from experiments across the world have the potential to reduce animal use and to make valuable samples immediately available for analysis when new technologies become cost-effective. Given that the costs of animal use are likely to rise and the costs of analysis decline, this would seem to be an efficient use of resources. However, establishing usable biobanks will require a marked change in the approach to our experiments [25, 26]. Firstly, experiments will need to be designed not just around current hypotheses but around future ones. Since it will be unusual to be able to identify future hypotheses, the more likely approach will be to take as many samples as possible, whether or not they have been identified as important within the specific hypotheses behind the experiment. Secondly, multiple replicates of specific samples will need to be taken, in order to allow subsets of samples to be allocated to more than one technique while still retaining replicate samples for future analysis. Thirdly, sufficient long-term storage needs to be available to maintain sample biobanks for prolonged periods under appropriate conditions. Fourthly, experiment, animal and sample metadata need to be maintained in a form which is easily accessible and transferable to analytical software. Finally, we will need processes for making such sample biobanks widely available.

### Planning biobanks: horizon scanning

Efficient design of experiments which can test current hypotheses but also generate bias-free data from existing omics technologies, as well as appropriate samples to test future hypotheses and supply future technologies, will require a high level of ‘horizon-scanning’ by animal scientists. Since new technologies are usually extremely costly when first developed and become progressively cheaper over time (Fig. 1), there is usually a relatively long lead time between a particular technique becoming visible in the scientific literature and becoming sufficiently well established, reproducible and cheap to consider on large biobanks of samples.

One specific area in which horizon-scanning is important is the analysis of the role of microbiomes and their role in human and animal health and disease. The progress described above, from identifying a small number of organisms by conventional culture to larger numbers of organisms by DNA-based approaches, still only returns a list of the resident microbiota. It is increasingly apparent that these organisms need to be considered as a series of interacting ecosystems within the host. Understanding the way in which these ecosystems respond to the environment (diet, pro/prebiotics, host immunity, etc.) and subsequently manipulate the host immune and metabolic systems will require extensive use of metagenomic, transcriptomic, proteomic and metabolomic data. Since microbiomes occupy spatially distinct compartments within the host (lumen, mucosa; small and large intestine, caecum and colon), techniques capable of applying these omics technologies at high resolution will also be needed. Researchers will need to be aware of what technologies are likely to become realistically available over the next few years as they design experiments.

**Fig. 6:**
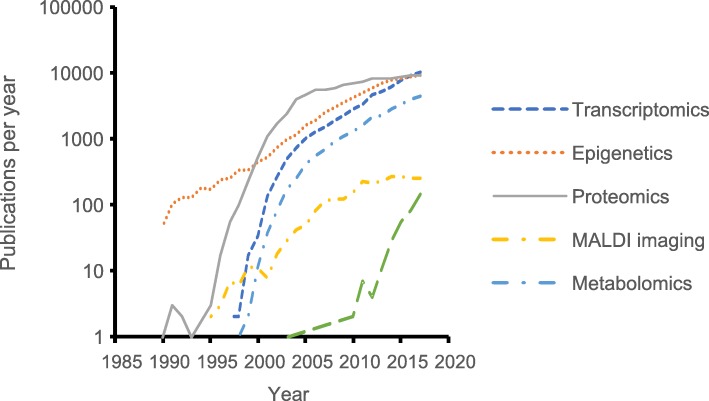
Numbers of publications where the title, abstract or keywords include terms associated with transcriptomics, epigenetics, proteomics, MALDI imaging, metabolomics or single cell transcriptomics. Source: Web of Knowledge (Clarivate Analytics), December 2018

It is already possible to estimate the rate of uptake of different technologies by carrying out literature searches using appropriate terms (Fig. 6). There are methodological limitations associated with this, since it is reliant more on the use of particular terms by authors than on the actual use of the technology, but searching for terms associated with proteomics, transcriptomics, metabolomics and single cell transcriptomics suggests that the interest in their use began in 1996, 1999, 2000 and 2013, respectively and grew approximately 3-fold every year to begin with. The apparent slowing of uptake is likely to be a consequence either of techniques becoming fully adopted or of specific terms no longer being used in publications once they become widely adopted. Proteomics, transcriptomics and metabolomics on tissue or biofluid samples are now relatively mature techniques with clear requirements for sample preparation and storage, and appropriate samples would be relatively easy to take routinely from all experiments. In contrast, single cell transcriptomics is still in its infancy, but the pattern of growth of publications suggests that it will rapidly become as important as the previous three. It may be that routine sampling into biobanks should include cell suspensions from enzymatically disrupted tissues stored in liquid nitrogen for future sorting and analysis. In contrast, references to epigenetics or matrix assisted laser desorption ionisation (MALDI) imaging suggest continuous but slower growth in interest, possibly due to differences in the way the costs of the technologies have changed. However, storage requirements for these techniques are also well established and appropriate samples could be incorporated into sample retrieval protocols.

Future technologies which are likely to become available at a cost which enables their routine use on new and biobanked samples include:

- MALDI imaging provides unparalleled access to peptides and small organics in tissues at current resolutions of around 20 μm, and can be used to build up 2- and 3-dimensional maps of function within tissues [27, 28]. The cost of processing tissues is currently very high, which has probably slowed its uptake (Fig. 4), but flash-frozen tissues or samples taken for routine cryosectioning are entirely appropriate for future analysis
- Lipidomics and glycomics, particularly for nutritional studies and for analyses of microbiomes, are likely to become more widely used [29]. Currently, samples preserved conventionally (flash freezing) are also appropriate for these techniques
- Techniques for culturing currently unculturable eukaryotes are under development, largely based on identifying missing metabolic pathways from whole genome sequencing [30]. Once these become available, it will be possible to examine the function of specific organisms within complex ecosystems (rather than just their relative abundance). While this may be possible from flash frozen samples, it may also be that specific transport media are required, which may make these techniques unavailable on current samples.
- Single cell eukaryotic transcriptomics and, more distantly, prokaryotic transcriptomics is now contributing significantly to understanding tissue biology by demonstrating the variation between cells rather than the average cell [31, 32]. Significant advances in retaining viability of cryopreserved cells mean that it may be possible to isolate and store cells from experimental tissues, and carry out single-cell transcriptomics at a later date. In contrast, single cell transcriptomics of bacterial cells is still technologically challenging and may or may not be possible on currently archived samples.
- Digital polymerase chain reaction (PCR) and a range of other approaches to targeted analysis of gene expression.
- Analysis of single nucleotide polymorphisms (SNP) or whole genomes of all experimental animals at the end of the experiment to provide an explanatory variable.

However, there are, inevitably, a set of techniques which are likely to become available in the future which are beyond the ability of horizon-scanning to prepare for. Many of these are techniques associated with generating samples or data from live animals. Examples include:

- Site-specific sampling of intestinal microbiomes using modified, orally-administered capsules such as those initially designed for targeted drug delivery or wireless endoscopy [33]. Such devices might incorporate a controlled release of a protein or nucleic acid stabiliser once the sample has been taken
- Rapid analysis of single nucleotide polymorphisms or whole genomes of all experimental animals in order to control for genetic variation during randomisation at the start of the experiment. Thus, for example, experiments frequently randomise genders into groups to control for known effects. Rapid SNP typing would allow randomisation of sire (for example, where pooled batches of semen have been used for insemination) and of other loci with known or probable effects.
- Gene editing of animals to create new tools (for example, cell-lineage-specific fluorescence *in vivo*) or to establish causal relationships (for example, knockouts or knockdowns of viral receptor proteins) [34]. The widespread adoption of competitive, regularly interspaced short palindromic repeats (CRISPR/Cas9) technologies is likely to make this type of approach much more widely usable in future. Recent developments have enabled editing of multiple genes in a single process, simplifying the previous approach of crossing and backcrossing strains [35]
- Serial imaging of experimental animals using, for example, high energy magnetic resonance imaging (MRI) or multiple-photon microscopy to identify changes in internal organ structure or body composition [36]. While the current resolution of such devices requires some form of restraint (sedation or anaesthesia) increased power may make serial imaging of conscious animals possible.

As these techniques, and those which are genuinely unforeseen, come into routine use, we can expect them to be genuinely disruptive, necessitating repeated experiments. However, these developments are likely to be much further off, and should not prevent us from dealing with the more immediate methodologies which are relatively easy to prepare for.

### Governance

Although the potential benefits of biobanks of samples from animal experiments are apparent, establishing processes for governance of samples and data may present continued problems. Again, this area has been widely explored in human medicine [26, 37, 38]. Specifically, there is a need to establish scientific review boards capable of assessing requests for access to biobanked material. Such review boards will need to be able not only to assess the specific value of each request considered in isolation (is it asking an appropriate question? is the proposed methodology suitable?), but also against the wider scientific value of the samples (would it be better to wait for a better technology? would it be better if the samples were combined with those from another experiment, perhaps from another institution?). Such review panels have been appropriate for large cohort studies in human medicine, where the size of the biobank makes an individual panel appropriate, but the kind of controlled intervention study more common in animal science will make individual panels difficult to establish, placing the responsibility on the institutes rather than the individual.

Both charity and government funders are taking the view that the outcomes and results of publicly-funded research should be publicly available, rather than ‘owned’ by individual researchers or institutes. The animal science community is likely to come under pressure to resolve these issues as part of this increasing trend towards open science. However, institutes will find it difficult to fund such resources internally [39], and external funders also need to be aware of the costs of the maintenance and governance of biobanks. This requires investment, which is the primary reason why such biobanks are still relatively infrequent in animal science. In human medicine, long-term cohort studies have become an important resource for novel research using technologies which were completely unavailable when the studies were initially funded [37, 40]. Funders of medical research are now aware of the value of such biobanks, and we need reviewers and funders of animal science to adopt the same view.

An important consideration for funders should be the effect of biobanking on the value of individual samples. Thus, one could consider that the value of a sample biobank on which no sample processing to data has been carried out is entirely potential. Once a specific technological approach has been used on a subset (for example microbial metagenomics of caecal and colonic contents), those data and the inferences from it should become available as metadata. The value of the remaining samples then increases, since subsequent analysis of mucosal or hepatic transcriptome, for example, can be linked back to the large intestinal microbiome. As more analyses are carried out, the samples and the data from them become increasingly valuable, providing that they are made freely available as part of the metadata. Again, this has been recognised in human clinical trials, where sharing of data may be required for registration of the experimental design [41].

### Replacement, reduction and refinement (the 3Rs)

As discussed, ethical considerations and the increasing costs of animal production will result in pressure on the use of animals in research, even where experiments are designed to answer questions around livestock agriculture. We can expect increasingly detailed examination by funding agencies of power calculations, and increasing expectation that experimental designs take the 3Rs into consideration [42]. This is likely to result in pressure in all of the areas discussed above. Firstly, it seems likely that it will become difficult to justify repeating experiments solely to acquire new samples. Under these circumstances, the establishment of biobanks will clearly contribute to the aims of the 3Rs and should be seen as ethically desirable.

Secondly, experimental designs will need to take considerably more account of full or stratified randomisation algorithms [43]. Clearly, where an experiment is designed with two intervention arms (e.g. postweaning probiotic feeding and control), one approach would be simply to randomise piglets at weaning into two groups. However, if we have prior evidence for maternal or gender effects on microbiome, it may be more appropriate to stratify our randomisation to ensure full litter and gender balance between the two groups rather than to assume that full randomisation will achieve this. In addition, stratification allows variation due to gender and litter to be partitioned by adding them as fixed factors in the final analysis, whereas in the fully randomised design, variation due to these factors appears in the error term, reducing the power of the experiment. Similarly, for microbiome or infectious disease experiments, animals penned together are likely to share microorganisms such that animals in a pen are no longer independent and pen becomes the experimental unit rather than animal [44]. Both of these will affect experiment size and consequent costs.

Finally, it will be increasingly important to estimate the power of experiments as accurately as possible in order to use appropriate numbers of animals. Current approaches to power analysis are limited to relatively simple experimental designs and are not good with estimating numbers necessary to identify observations. In general, effective power calculations under these conditions rely on the use of simulated data, but these algorithms need to be extended to make estimating effect sizes more intuitive, particularly where multiple factors have been used to stratify animals as above [45, 46].

## Conclusions

The overall costs of animal experiments are unlikely to fall significantly in future. Pressure to replace, reduce and refine the use of animals in experiments will make it more difficult to repeat experiments which have already been carried out, simply in order to access samples for new technological advances. In contrast, the costs of processing samples through existing pipelines are likely to continue to decrease and new technologies are likely to become affordable. As animal scientists, we have a responsibility to design our experiments to be as future proof as possible by collecting far more samples than we require to test our initial hypotheses and storing them in biobanks in such a way that they can be used for testing novel or linked hypotheses in future. This will require a considerable shift in attitudes to experiments: we will need a culture of horizon-scanning for technologies likely to be usable in the near future. We will need clear, consistent archiving of samples and metadata. Most importantly, we need to understand the value of samples taken from our animal experiments, and the extent to which that value increases as they are analysed.

## Abbreviations

3Rs: Replacement, reduction, refinement
CRISPR: Competitive, regularly interspaced short palindromic repeats
DGGE: Denaturing gradient electrophoresis
DNA: Deoxyribonucleic acid
MALDI: Matrix assisted laser desorption ionisation
MRI: Magnetic resonance imaging
NIH: National Institutes of Health
OTU: Operational taxonomic unit
PCR: Polymerase chain reaction
rRNA: ribosomal ribonucleic acid
SNP: Single-nucleotide polymorphism
